# *Kharon1* Null Mutants of *Leishmania mexicana* Are Avirulent in Mice and Exhibit a Cytokinesis Defect within Macrophages

**DOI:** 10.1371/journal.pone.0134432

**Published:** 2015-08-12

**Authors:** Khoa D. Tran, Danielle P. Vieira, Marco A. Sanchez, Jessica Valli, Eva Gluenz, Scott M. Landfear

**Affiliations:** 1 Department of Molecular Microbiology & Immunology, Oregon Health & Science University, Portland, Oregon, United States of America; 2 Sir William Dunn School of Pathology, University of Oxford, Oxford, United Kingdom; University at Buffalo, UNITED STATES

## Abstract

In a variety of eukaryotes, flagella play important roles both in motility and as sensory organelles that monitor the extracellular environment. In the parasitic protozoan *Leishmania mexicana*, one glucose transporter isoform, LmxGT1, is targeted selectively to the flagellar membrane where it appears to play a role in glucose sensing. Trafficking of LmxGT1 to the flagellar membrane is dependent upon interaction with the KHARON1 protein that is located at the base of the flagellar axoneme. Remarkably, while **Δ** *kharon1* null mutants are viable as insect stage promastigotes, they are unable to survive as amastigotes inside host macrophages. Although **Δ** *kharon1* promastigotes enter macrophages and transform into amastigotes, these intracellular parasites are unable to execute cytokinesis and form multinucleate cells before dying. Notably, extracellular axenic amastigotes of **Δ** *kharon1* mutants replicate and divide normally, indicating a defect in the mutants that is only exhibited in the intra-macrophage environment. Although the flagella of **Δ** *kharon1* amastigotes adhere to the phagolysomal membrane of host macrophages, the morphology of the mutant flagella is often distorted. Additionally, these null mutants are completely avirulent following injection into BALB/c mice, underscoring the critical role of the KHARON1 protein for viability of intracellular amastigotes and disease in the animal model of leishmaniasis.

## Introduction

*Leishmania* and *Trypanosoma* are kinetoplastid parasitic protozoa that cause devastating diseases affecting millions of people worldwide [1, 2]. The disease-causing intracellular *Leishmania* amastigotes reside and proliferate within phagolysosomal vesicles inside mammalian macrophages (reviewed in [3, 4]). The determinants that allow amastigotes to thrive within the hostile environment of the phagolysosome are poorly understood, although a number of molecular genetic studies have identified some genes whose deletion can be tolerated by promastigotes but lead to impaired viability of intracellular amastigotes [5–7].

The trypanosomatid flagellum is recognized as an important organelle required for cell motility and in some cases for cell division during replication [8, 9]. More recent studies strongly suggest that flagella in *Leishmania* and trypanosomes are also likely to serve a sensory role and transmit information about the extracellular environment into the cell interior, similar to the role of sensory cilia and flagella in other eukaryotes [10, 11]. For example, the flagellar-specific aquaporin AQP1 of *L*. *major* is required for sensing osmolarity of the medium [12], and the flagellar glucose transporter from *L*. *mexicana*, LmxGT1, probably participates in sensing glucose in the medium and mediating the transition from logarithmic to stationary phase of growth [13]. Recent proteomic analyses of the *T*. *brucei* flagellum [14, 15] identified a host of proteins, many with structures suggesting possible roles in sensing or signal transduction, that are localized to the flagellar membrane of bloodstream and procyclic form parasites.

*Leishmania* intracellular amastigotes possess a short non-motile flagellum that resembles a sensory cilium in structure [16], suggesting that it could be involved in monitoring the environment of the macrophage phagolysosome. Furthermore, the tip of this short amastigote flagellum forms a close connection with the vacuolar membrane of the host macrophage [16, 17], and it has been suggested that this may constitute a putative parasite ‘synapse’ that might be required for sensory perception or for delivery of parasite proteins to the macrophage. Thus flagellar membrane proteins that serve as sensors or that mediate synapse formation may be crucial for parasite survival inside mammalian hosts.

Although the cell body and flagellar components of the plasma membrane are physically contiguous, the existence of proteins that are restricted to one or the other membrane [5] demonstrates that these two domains of the cell surface are distinct. However, the mechanisms for selectively targeting membrane proteins to each domain are obscure. We have previously described the flagellar localization of the *L*. *mexicana* Glucose Transporter 1, LmxGT1 [5, 18] and demonstrated that flagellar targeting of this permease is dependent upon a sequence located within the unique N-terminal domain of the protein [18]. Subsequently, we employed formaldehyde crosslinking followed by tandem affinity purification and mass spectrometry to identify a trypanosomatid-specific protein, named KHARON1 (KH1), which interacts with the flagellar targeting domain of LmxGT1 and is required for targeting the permease to the parasite flagellum [7]. Notably, although **Δ** *kh1* mutants were fully viable as promastigotes and were able to infect THP-1 derived macrophages, they were unable to survive inside the host cells and were cleared over the course of 7 days. These results suggest that KH1, while dispensable in promastigotes, is absolutely critical during the amastigote stage of the parasite life cycle.

In the current study, we have further investigated the requirement for KHARON1 in infectious amastigotes, both in macrophages in vitro and in the murine model for leishmaniasis. Remarkably, **Δ** *kh1* intracellular amastigotes replicate nuclei, kinetoplasts, and flagella but fail to undergo cytokinesis and accumulate as multinucleate cells with altered morphology before expiring. The lethal phenotype is not exhibited by extracellular axenic amastigotes, indicating a critical role for KHARON1 specifically in amastigotes residing in the intracellular environment. Furthermore, the amastigote-lethal phenotype is also apparent following injection into BALB/c mice, as the **Δ** *kh1* null mutants are largely or completely avirulent in this murine model of infection.

## Materials and Methods

### Parasite cultures, transfection, and differentiation

Wild type *Leishmania mexicana* promastigotes (strain MNYZ/BZ/62/M379) were cultured in RPMI 1640 medium (Invitrogen, Carlsbad, CA, USA) supplemented with 10% heat-inactivated fetal bovine serum (FBS) (Thermo Scientific Hyclone, Logan, UT), 0.1 mM xanthine, and 5 **μ**g/mL of hemin. **Δ** *lmxkh1* (**Δ** *kh1*) null-mutants [7]were maintained in RPMI or RPMI supplemented with 50 **μ**g/mL of puromycin and 50 **μ**g/mL of phleomycin (InvivoGen, San Diego, CA, USA). The **Δ** *kh1* null-mutant add-back line, **Δ** *kh1*[int*Kh1*], was maintained in RPMI supplemented with 50 **μ**g/mL of puromycin and 25 **μ**g/mL of blasticidin. All promastigote cultures were maintained at 26°C. *Leishmania* promastigotes were transfected according to previously described electroporation techniques using a Bio-Rad Gene Pulser Xcell [19].

For differentiation into axenic amastigotes, approximately 5 x 10^7^ promastigotes were seeded into 5 mL of a base culture medium described previously [20] adjusted to pH 5.66 at 22°C and maintained at 32°C. The cultures were incubated for 10 days to allow for transformation, and the axenic amastigotes were then passaged weekly.

### Generation of add-back line from Δ*kh1* mutant parasites by re-integration of *Kh1* into its endogenous locus

The *Kh1* ORF was fused to the *Thosea asigna* virus 2A peptide (2A), *Renilla* luciferase (Luc), and the blasticidin resistance (BSD) protein, in that order, and then flanked by sequences immediately upstream and downstream of the *Kh1* ORF as described previously [7]. The linearized construct was transfected into **Δ** *kh1* mutant promastigotes, and transgenic parasites containing the integrated transgene replacing the integrated phleomycin drug resistance cassette were selected on plates containing 80 **μ**g/mL blasticidin and 50 **μ**g/mL of puromycin. The 2A peptide induces a co-translational intra-ribosomal cleavage during synthesis of the fusion protein [21], so that a KH1::2A fusion protein, unattached to Luc::BSD, is generated in these add-back parasites.

### Growth curves of promastigotes and axenic amastigotes

Promastigote growth curves were determined by assessing culture density over a 7-day time-course. Ten milliliter cultures where inoculated with 1 x 10^6^ cells (1 x 10^5^ cells/mL) and cell counts were determined each day using a hemacytometer. For axenic amastigote growth curves, 5 mL cultures were inoculated with 2.5 x 10^6^ cells (5 x 10^5^ cell/mL), and cell counts were taken at two-day intervals during a 16-day time-course. At least three growth curves, each counted in triplicate, were performed for promastigotes and axenic amastigotes.

### Molecular markers and immunodetection

For immunofluorescence, parasites were collected, washed once with PBS, and attached to poly-L-lysine-treated coverslips for 20 min. Cells were fixed and permeabilized with methanol at -20°C for 8 min. For P4 endonuclease staining, the parasites were fixed with 2% formaldehyde (Ultra Pure EM grade, Polysciences, Warrington, PA, USA), for 5 min at 4°C and permeabilized with 0.05% Triton X-100 for 5 min. Fixed cells were washed 3 X 5 min with PBS, then blocked with 5% normal goat serum for 20 min. Parasites were incubated with primary antibodies for 1 h at room temperature, washed 5 X 10 min with PBS, and then incubated with secondary antibodies for 1 h. After secondary antibody incubation, cells were washed as described above. Coverslips were mounted onto microscope slides using DAPI Gold Prolong reagent (Molecular Probes, Eugene, OR, USA). Antibodies, dilutions, and sources are as follows: rabbit **α**-tubulin, 1:500 (Sigma-Aldrich, St. Louis, MO, USA); mouse **α**-tubulin, 1:2000 (Sigma-Aldrich); mouse P4, 1:200 (a generous gift from Diane McMahon-Pratt, Yale University School of Public Health, New Haven, CT, USA; [22]); rabbit 2A (TaV2A), 1:200 (Millipore, Burlington, MA, USA); rat YL1/2, 1:500 (Millipore); anti-rabbit IgG Alexa-488, 1:1000 (Molecular Probes); anti-mouse IgG Alexa-594, 1:1000; anti-rabbit IgG Alexa-594, 1:1000; anti-rat IgG Cy3 1:500 (Jackson ImmunoResearch, West Grove, PA, USA).

For immunoblotting, cells were collected and washed with 1x PBS. Cells were lysed in 1x lithium dodecyl sulfate (LDS) sample buffer (Invitrogen) and loaded onto 4–12% polyacrylamide gels for electrophoresis (Invitrogen). Proteins were then transferred onto nitrocellulose membranes, blocked with 5% milk in PBS containing 0.1% Tween-20, and probed with primary antibodies in blocking solution. Antibodies, dilutions, and sources are as follows: mouse P4, 1:50; and mouse **α-**tubulin, 1:5000. Anti-mouse and anti-rabbit HRP-conjugated secondary antibodies were used at 1:20,000 (Thermo Scientific Pierce) with 5% milk in PBS containing 0.1% Tween-20.

### Deconvolution microscopy

Fluorescence images were captured on a Deltavision Image Restoration System (Applied Precision, Issaquah, WA, USA) consisting of a Nikon Eclipse TE2000 microscope base, mercury light source, Applied Precision light homogenizer and Nanoposition XYZ stage and a Kodak CH350 CCD. Cells were imaged at room temperature through a 60x 1.40NA Nikon objective using SoftWoRx acquisition software version 5.0.0-R6 (Applied Precision, Issaquah, WA, USA). Images were deconvolved in SoftWoRx and then analyzed and processed using ImageJ (NIH, Bethesda, MD, USA). Figures were constructed using Adobe Illustrator CS6 (Adobe Corporation, San Jose, CA, USA).

### Macrophage infections

The human acute leukemia monocyte cell line (THP-1) was cultivated in RPMI 1640 medium (Invitrogen) supplemented with 10% heat-inactivated FBS (Thermo Scientific), 25 mM HEPES, 1% L-glutamine, 50 mM glucose, 5 mM sodium pyruvate, and 1% streptomycin/penicillin at 37°C and 5% CO_2_. The cultures were diluted every 3 days to prevent cell count from exceeding 1 x 10^6^ cell/mL. Cells were kept for a maximum of 20 subculture dilution cycles. THP-1 cells were differentiated with 100 ng/mL of phorbol 12-myristate 13-acetate (Sigma) for 48 h at 37°C, 5% CO_2_. Differentiated THP-1 cells are adherent and were seeded in 4-well Lab-TekII Chamber Slides (Nalge Nunc International, Rochester, NY) at a confluence of 3 x 10^5^ cells per well. *L*. *mexicana* promastigotes at stationary phase were added to the plates (10:1 promastigotes:macrophage ratio) for 4 h. Cultures were then washed several times until the majority of extracellular promastigotes have been removed. The 4 h time point was taken after the washes were complete and the other infections were incubated for 1 day, 3 days, 5 days, and 7 days at 37°C in a 5% CO_2_ environment. *L*. *mexicana* axenic amastigotes used in infection experiments were added at a 3:1 amastigotes:macrophage ratio unless otherwise indicated. Slides were stained using the HEMA3 STAT PACK staining kit as described by the manufacturer (Fisher Scientific, Kalamazoo, MI, USA). Infected macrophages were examined using a Nikon Eclipse 50i microscope equipped with a 100x 1.25NA oil objective (Nikon Instruments, Melville, NY, USA), and images where captured using a white Samsung Galaxy S4 equipped with a CMOS 13 mega-pixel digital camera (Samsung USE, Ridgefield Park, NJ, USA). The number of parasites per 100 macrophages was determined by counting 300 cells in each of the triplicate experiments per round of infection.

### Purification of intracellular amastigotes

Infected THP-1 macrophages were grown in 75 cm^2^ flasks (6x10^6^ macrophages/flask) and intracellular amastigotes were purified as follows for each flask. Macrophage cultures were washed with ice-cold PBS containing 2 mM EDTA. Macrophages were scraped off of the surface of the flask using a cell scraper (SARSTEDT, Newton, NC, USA) in 5 mL of PBS + 2 mM EDTA. Cells were pelleted and resuspended in 1 mL of PBS. The cell suspension was passed through a 27-guage needle 5 times, followed by 10–20 passes through a 30-guage needle until 99% of macrophages have been lysed. Subsequently, the cell suspension was brought up to a 15 mL volume and pelleted at 1,000 g. The cell pellet was resuspended in 500 **μ**L of PBS + 2mM EDTA and layered onto 500 **μ**l 90% PERCOLL (Sigma), followed by centrifugation at 21,000 g for 10 minutes in a micro-centrifuge. Amastigotes were collected at the interface and transferred to a new 1.5 mL tube.

### Mouse infections, lesion measurements, and parasite load

For passaging of parasites through mice (BALB/c strain, female, 6–8 weeks old, Jackson Laboratories, Bar Harbor, ME, USA), animals were infected by injecting the hind footpad with 5 x 10^6^ stationary phase promastigotes. Footpad tissue was removed at the indicated time, homogenized, and transferred to RPMI medium (wt), RPMI medium containing 50 **μ**g/mL of puromycin and 50 **μ**g/mL of phleomycin (**Δ** *kh1*), and RPMI medium containing 50 **μ**g/mL of puromycin and 25 **μ**g/mL of blasticidin (**Δ** *kh1*[int*Kh1*]) to allow outgrowth of animal-passaged promastigotes. Footpads were harvested from wild type and **Δ** *kh1*[int*Kh1*] infections 4 weeks after injection. The **Δ** *kh1* parasites were harvested 2 weeks after infection, because no mutant parasites could be recovered at either 3 or 4 weeks post-injection. Subsequently, groups of 5 mice were inoculated with 5 x 10^6^ stationary-phase animal-passaged promastigotes in the right footpad in 20 **μ**l of PBS. The thickness of each footpad was measured weekly with a Vernier caliper, and the dimension at week 0 was subtracted from each subsequent measurement to calculate the lesion size. After 8 weeks, the experiment was terminated and footpads were dissected and homogenized in 4 ml of RPMI. An estimation of parasite load was achieved by limiting dilutions using 96-well flat-bottom microtiter plate as described previously [23] and visualized on an inverted light microscope. If no parasites were detected at any dilution within a dilution series, then estimated parasite load = 0. If parasites were detected in a dilution series, then parasite load was estimated by using the highest dilution at which promastigotes could be detected after 10 days of incubation at 26°C.

All experiments with animals were approved by the Institutional Animal Care and Use Committee of the Oregon Health & Science University, approval number IS00000553, and followed guidelines of the Association for Assessment and Accreditation of Laboratory Animal Care. Anesthesia was achieved by intraperitoneal injection of ketamine, 90 mg/kg, and xylazine, 10 mg/kg. Euthanasia was carried out by exposing mice to a saturated atmosphere of isoflurane.

### Electron microscopy

THP-1 macrophages infected with wild type, **Δ** *kh1*mutants, and **Δ** *kh1*[int*Kh1*] add-back axenic amastigotes (at a ratio of 10 amastigotes:1 macrophage) were harvested at 1 day, 2 days, and 3 days post infection, fixed in 2.5% glutaraldehyde in culture medium, and incubated at room temperature for 3 minutes. Fixed cells were subsequently collected and centrifuged for 10 minutes at 350 x g. The cells were resuspended in 1 ml of buffered fixative (2.5% glutaraldehyde and 2% formaldehyde in 100 mM sodium phosphate buffer pH 7.4), pelleted for 10 minutes at 350 x g in screw-cap microtubes, and stored in fresh, buffered fixative.

Samples were washed in 0.2 M sodium phosphate buffer and then incubated at 4°C for 1 h with 1% aqueous osmium tetroxide in 0.1 M sodium phosphate buffer. Samples were subsequently washed with double distilled H_2_O (ddH_2_O) and then incubated in the dark at 4°C for 2 h in 2% aqueous uranyl acetate. Samples were once again washed with ddH_2_O prior to dehydration through a graded series of ethanol and embedded in Agar 100 epoxy resin. Axenic amastigotes were prepared essentially the same way, but because the cells did not form a firm pellet they were embedded in 4% agarose before staining with uranyl acetate to avoid loss of cells during the subsequent preparation steps. Sections (90 nm) were cut on an Ultracut 7 microtome (Leica), post stained with lead citrate and imaged in a Tecnai 12 Transmission Electron microscope (Hillsboro, OR, USA) at 120 kV. Images were captured with a Gatan Ultrascan 1000 CCD camera using SerialEM (bio3d.colorado.edu) and processed in ImageJ.

### Statistical analysis

Two-tailed *t*-tests were used to determine the significance of parasite load differences in and of the numbers of multi nucleated-intracellular amastigotes.

## Results

### KHARON1 localizes to the flagellum and pellicular cytoskeleton in amastigotes

Previous studies demonstrated that the KHARON1 protein (KH1) interacts with the flagellar targeting signal of the glucose transporter LmxGT1 and the **Δ** *kharon1* null mutant (**Δ** *kh1*) is deficient in trafficking LmxGT1 to the flagellar membrane [7]. Additionally, these null mutants were able to infect macrophages but the intracellular parasites did not replicate and died over a course of 7 days [7]. These observations strongly suggest that KH1 is expressed in amastigotes and that this protein has an essential function in that stage of the parasite life cycle.

To demonstrate expression of KH1 in amastigotes, we employed a previously described cell line in which a *Kharon1* (*Kh1*) open reading frame (ORF), epitope tagged with 3xHA (HA_3_) and the TaV2A (2A) peptide on its COOH terminus (KH1::HA_3_::2A), had been integrated into the endogenous *Kh1* locus [7]. The *Kh1*::*HA*_*3*_::*TaV2A*::*Luc*::*BSD* construct (Luc, *Renilla* luciferase ORF; BSD, blasticidin resistance gene) employed for integration contained a blasticidin resistance gene to allow genetic selection for integration and a TaV2A peptide that is cleaved co-translationally [21] to produce separate KH1:: HA_3_::2A and Luc::BSD polypeptides. This construct retains the 5’- and 3’-untranslated regions (UTRs) of the *Kharon1* mRNA and thus promotes expression of KH1 protein at near wild type levels under the control of the endogenous UTRs.

**Fig 1:**
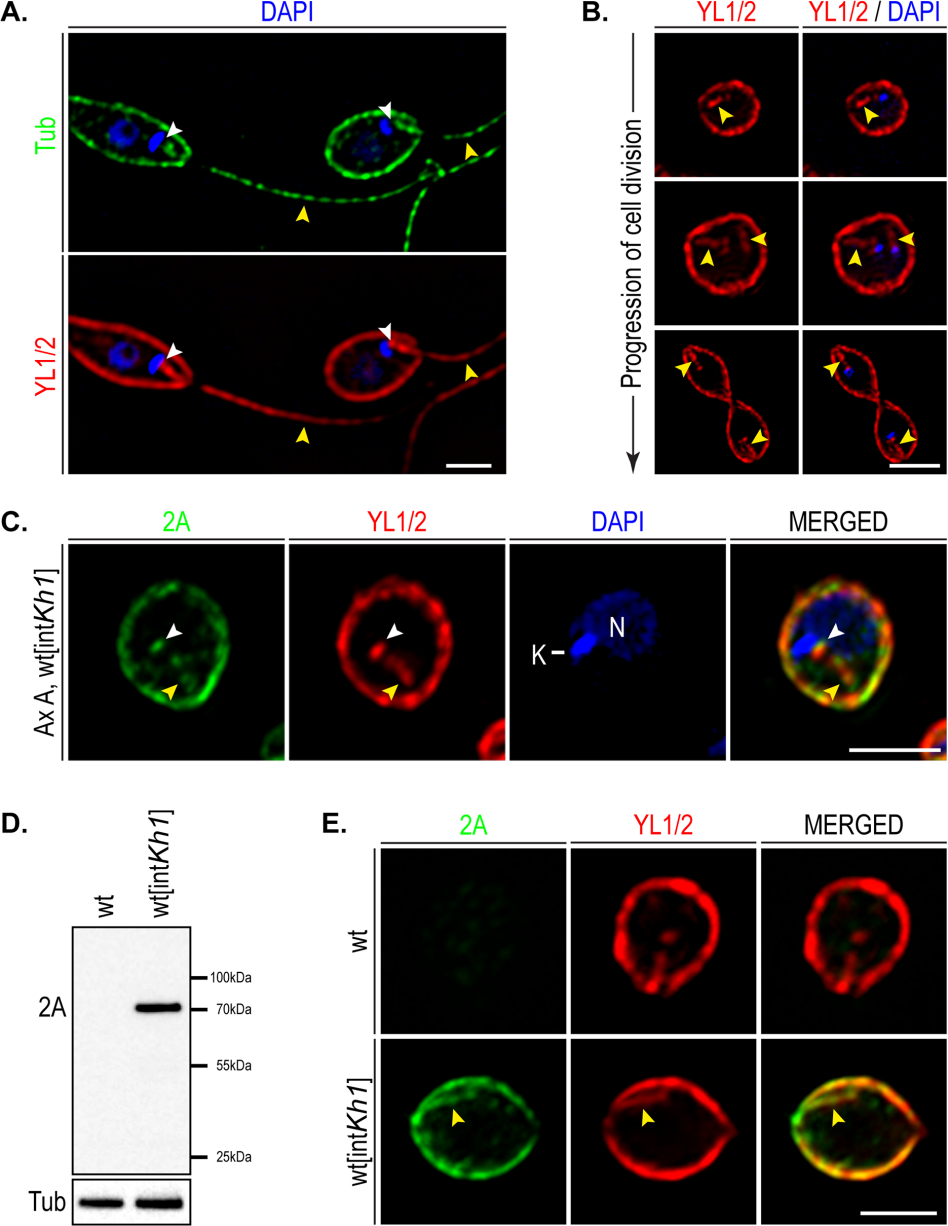
KHARON1 localization in *Leishmania mexicana* amastigotes. Scale bars, 3 **μ**m. A. *Leishmania mexicana* promastigotes were stained with antibody against **α**-tubulin (green), with the YL1/2 antibody against tyrosinated **α**-tubulin (red), and DAPI (blue). The basal body, which is anterior to the kinetoplast (white arrowhead), and the tubulin cytoskeleton are labeled by YL1/2. The yellow arrowheads indicate flagella. B. Cell division stages of axenic amastigotes stained with the YL1/2 antibody (red) and DAPI (blue). The amastigote flagellar structures are labeled with YL1/2 (yellow arrowheads). The kinetoplast is shown in blue, while the nuclei are out of focus in these sections. C. Co-localization of KH1::2A (green) and YL1/2 antibody (red) in axenic amastigotes. K indicates kinetoplast DNA, and N indicates the nucleus. The white arrowhead points to the base of the flagellum, and the yellow arrowhead points to the flagellar structure. D. Immunoblot of KH1::2A expression in purified intracellular amastigotes, and negative control (wild type amastigotes). E. Immunofluorescence of purified intracellular amastigotes, expressing KH1::2A from the *Kh1* locus, using antibody against 2A (green) and YL1/2 (red).

In promastigotes, the KH1::HA_3_::2A protein was localized to the basal body and the base of the flagellar axoneme that lies within the flagellar pocket and to the subpellicular microtubule network that subtends the plasma membrane, as revealed by immunofluorescence and immunoelectron microscopy [7]. Furthermore, KH1 tagged at either the N- or C-terminus produced the same pattern of localization, making it unlikely that the epitope tag causes artifactual mislocalization [7]. The localization of KH1 at the base of the flagellar axoneme in the region of the flagellar pocket was postulated to be important for its function in sorting the LmxGT1 glucose transporter from the flagellar pocket membrane into the adjacent flagellar membrane [7]. To evaluate localization of KH1::HA_3_::2A in amastigotes, we first sought an antibody that could stain efficiently various tubulin-containing structures, including the small amastigote flagellum that is almost completely contained within the flagellar pocket inside the cell body. The YL1/2 antibody [24, 25] that recognizes tyrosinated **α**-tubulin and potentially other cytoskeletal proteins [26] stained structures in both promastigotes (Fig 1A) and axenic amastigotes (Fig 1B) that appear to be i) the subpellicular microtubules at the periphery of the cell, ii) the flagellar axoneme, including an internal rod-like structure in the amastigote (Fig 1B and 1C) that initiates near the DAPI stained kDNA (blue, marked K in Fig 1C) and is present in two copies in cells where the kDNA has replicated (Fig 1B, middle panel), and iii) the basal body at the base of the flagellum (white arrowheads in Fig 1A and 1C). The ability of YL 1/2 to label multiple microtubule-based structures in *Leishmania mexicana* is similar to observations of a previous study employing another antibody directed against tyrosinated **α**-tubulin in which broad labeling of *L*. *donovani* microtubule containing structures was observed [27]. In contrast, two other antibodies directed against untryosinated **α**-tubulin (rabbit or mouse anti-**α**-tubulin, Sigma) did not efficiently label the amastigote flagellum (not shown), providing a compelling pragmatic reason to use the YL1/2 antibody instead.

Subsequently, immunofluorescence images of axenic amastigotes expressing KH1::HA_3_::2A, stained with the anti-2A antibody (Fig 1C), were collected. The green fluorescence representing the KH1::HA_3_::2A protein was present at the periphery of the cell, coincident with the YL1/2 staining subpellicular microtubules, and at an internal region (yellow arrowheads in Fig 1C) consistent with localization to the amastigote flagellum. Furthermore, axenic amastigotes expressing KH1::HA_3_::2A were used to infect THP-1 macrophages. After 5 days, intracellular amastigotes were purified and examined for KH1 expression. Immunoblots of lysates prepared from purified amastigotes were probed with the anti-2A antiserum and demonstrated that the KH1::HA_3_::2A tagged protein is expressed from the endogenous locus in intracellular macrophages (Fig 1D). Additionally, immunofluorescence of purified amastigotes expressing KH1::HA_3_::2A showed similar KHARON1 localization as in axenic amastigotes, while wild type amastigotes showed no specific labeling with the anti-2A antibody (Fig 1E).

Overall, these results indicate that the localization of KH1::HA_3_::2A is similar in promastigotes and amastigotes of *L*. *mexicana*. Moreover, the conserved location of this cytoskeleton-associated protein at the basal domain of the flagellum that is located within the flagellar pocket is consistent with its postulated function of translocating integral membrane proteins from the flagellar pocket membrane to the flagellar membrane in both life cycle stages. Thus KH1 located at the base of the flagellar axoneme could bind to flagellar targeting signals on membrane proteins destined for the flagellum [18], when each such protein is in the flagellar pocket membrane, and mediate its translocation into the adjacent flagellar membrane. The mechanistic details of such translocation must however await future studies.

### Generation of a Δ*kh1* add-back line complemented with an integrated copy of *Kh1*

**Fig 2:**
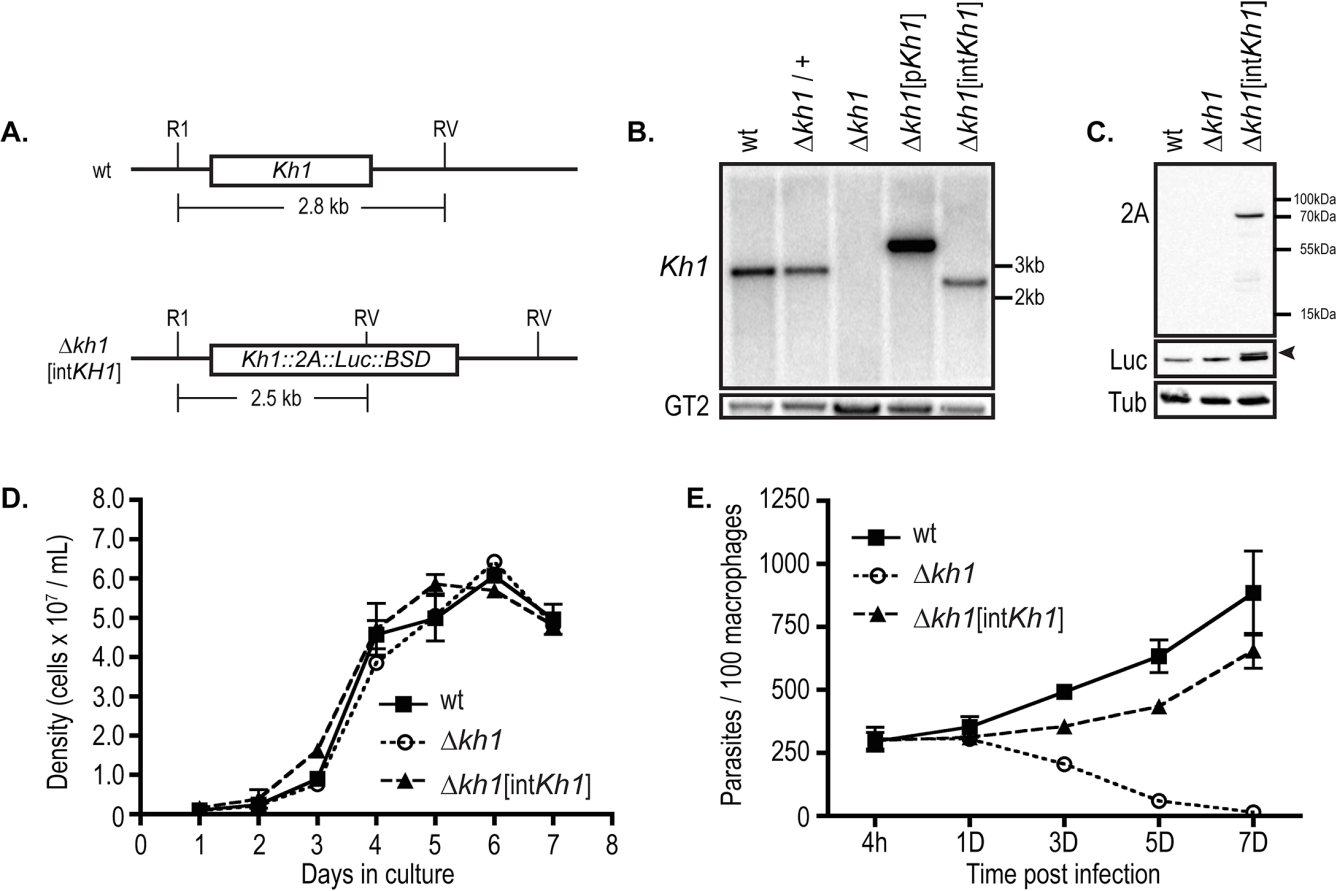
Rescue of ∆*kh1* mutants by genomic integration of a *Kh1* allele. A. Schematic representation of genomic region surrounding the *Kh1* gene in the wild type locus (top) and in the **Δ** *kh1*[int*Kh1*] add-back line in which a *Kh1*::2A::Luc::BSD fusion ORF was integrated into the gene locus (bottom). *Eco*R1 (RI) and *Eco*RV (RV) restriction sites are indicated along with the expected size of the digested genomic DNA (gDNA) fragment containing the *Kh1* open reading frame. B. Southern blot probed with the *Kh1* ORF (top) comparing *Eco*RI/EcoRV digested gDNA from the **Δ** *kh1*[int*Kh1*] add-back line to the **Δ** *kh1* mutants and an add-back line carrying an episomal copy of the *Kh1* ORF, **Δ** *kh1*[p*Kh1*] [7]. DNA from wild type (WT) and heterozygous knockout (**Δ** *kh1*/+) lines is also shown. The blot was stripped and re-probed with the *LmxGT2* ORF (GT2) as a DNA loading indicator. C. Immunoblot probed with antibody against the 2A epitope to detect the KH1::2A fusion protein. The Luc::BSD product is cleaved from the KH1::2A::Luc::BSD fusion and migrates at approximately 55 kDa (arrowhead), as demonstrated by probing the blot with antibody against luciferase (Luc). The slightly more rapidly migrating band present in each lane probably represents cross-reaction of the antibody with tubulin proteins (middle panel). A replicate blot was probed with anti-**α**-tubulin antibody (Tub) (bottom panel). D. Growth curve of cultured promastigotes comparing growth rate of wild type (wt), **Δ** *kh1*mutants and **Δ** *kh1*[int*Kh1*] add-back parasites over a 7-day time course. Data represent the average and standard deviation of 3 independent growth curves (n = 3). Each sample from each growth curve time point was counted in triplicate and averaged. E. Quantification of THP-1 infection with wild type, **Δ** *kh1*mutants, and **Δ** *kh1*[int*Kh1*] add-back promastigotes. Times post infection: h = hours, D = days. Data represent the averages and standard deviations of triplicate infections (n = 3), each counted in triplicate and averaged. These results are similar to those previously published for the **Δ** *kh1* null mutant complemented with an episomal copy of *Kh1* [7], but the current experiment confirms complementation by the re-integrated *Kh1* gene, the complemented line employed throughout this study.

To enable further characterization of **Δ** *kh1* mutants in amastigotes both in vitro and in animal models of infection, we generated an add-back line in which a *Kh1* gene was re-integrated into one allele of the disrupted *Kh1* locus. This stably complemented add-back line will express the KH1 protein at near wild type levels and will not undergo changes in the *Kh1* gene copy number during the extended periods of time required for animal experiments, a potential problem with episomal expression vectors. To this end, we re-integrated the *Kh1*::*TaV2A*::*Luc*::*BSD* construct into one of the disrupted *Kh1* endogenous gene loci in **Δ** *kh1* mutants to generate the **Δ** *kh1*[int*Kh1*] add-back line. Proper integration of this rescue cassette (Fig 2A) was verified by genomic Southern blotting (Fig 2B), and expression of the KH1::TaV2A protein (KH1::2A) was confirmed by immunoblotting using an anti-2A antibody (Fig 2C). Promastigotes of wild type, **Δ** *kh1* mutants, and **Δ** *kh1*[int*Kh1*] rescue cells grew with similar dynamics in culture (Fig 2D), as expected for a null mutant that does not affect viability of promastigotes [7]. Furthermore, **Δ** *kh1*[int*Kh1*] add-back promastigotes were able to complement the **Δ** *kh1* mutant avirulent phenotype during infection of cultured THP-1 derived macrophages (Fig 2E). Therefore, this stable add-back line is suitable for characterization of KH1 function in *Leishmania* parasites.

### Δ*kh1* mutants do not form lesions during mouse infections

**Fig 3:**
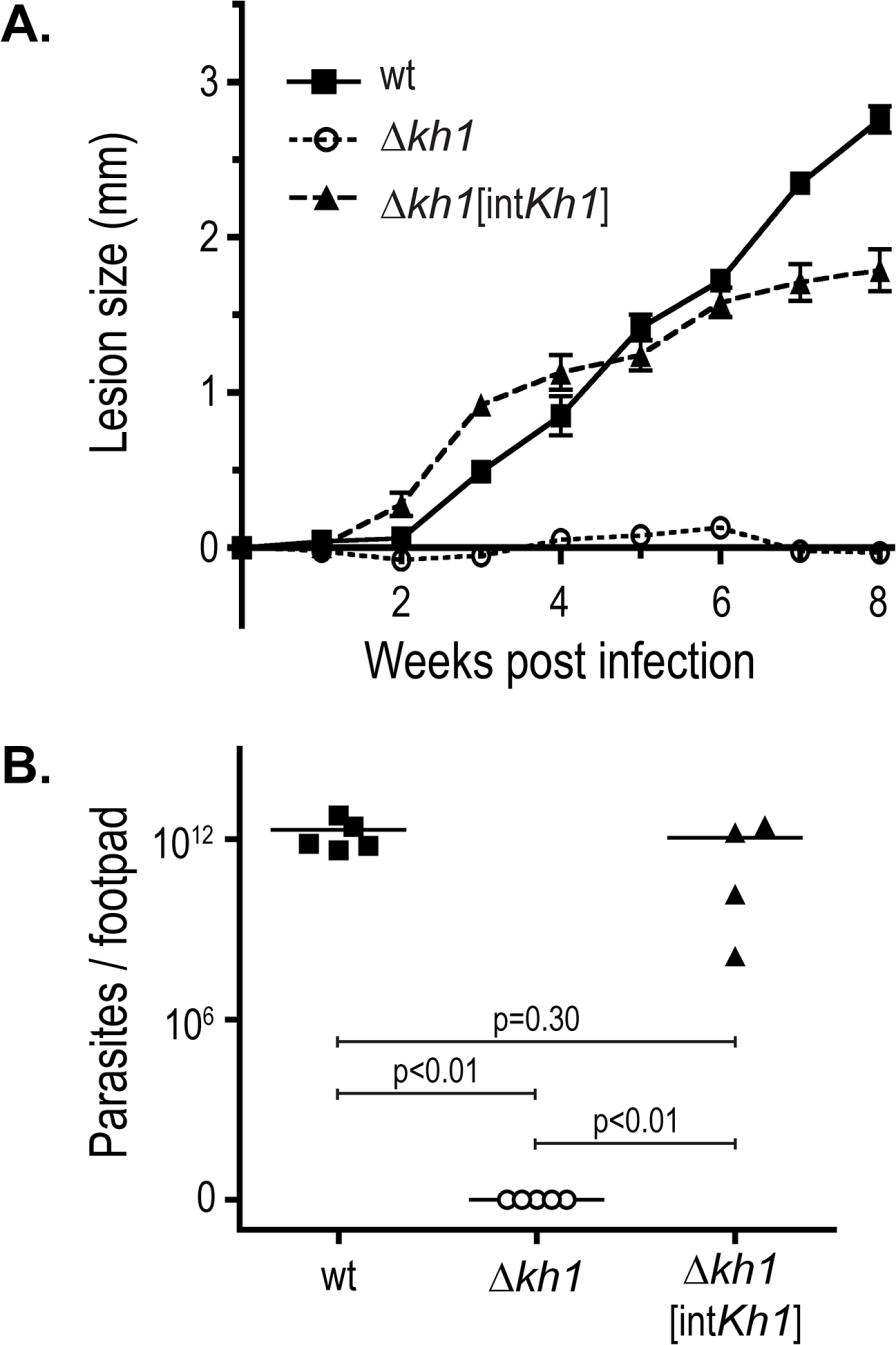
Infection of BALB/c mice with Δ*kh1* mutant and Δ*kh1*[int*Kh1*] add-back promastigotes. A. Average lesion size caused by wild type (n = 5 mice), **Δ** *kh1*mutants (n = 5), and **Δ** *kh1*[int*Kh1*] add-back (n = 4) *L*. *mexicana* parasites. One mouse (out of 5) inoculated with **Δ** *kh1*[int*Kh1*] add-back parasites did not form a detectable lesion, possibly due to failed injection, and therefore was excluded from this analysis. The slight difference in lesion kinetics between wild type and add-back parasites may be due to the fact that the add-back line encompasses only one, rather than two, copies of the *Kh1* gene. The data in the graph represent the average and standard deviations of measurements for all the footpads (n = 4 or 5) representing each strain. B. Estimated parasite load from limiting dilution experiments. Footpads were removed from 5 mice for the WT and **Δ** *kh1* strains and for 4 mice from the **Δ** *kh1*[int*Kh1*] strain at week 8 post infection. Eight limiting dilution replicates were done for each footpad, and the results were averaged to obtain a number for each footpad. The line in each graph represents the average estimated parasite load for all footpads representing that strain (2.5x10^12^ parasites per footpad for wild type and 1.3x10^12^ parasites per footpad for the **Δ** *kh1*[int*Kh1*] strain). These parasite burdens are similar to those observed for BALB/c mice infected with *L*. *amazonensis* under conditions that generated lesions of similar thickness [31]. The numbers and horizontal bars indicate the p values for comparisons of the data for relevant strains.

We have reported that **Δ** *kh1* mutants are unable to maintain infection of cultured THP-1 derived macrophages (Fig 2E and [7]). However, it is unknown whether the inability to survive in macrophages in vitro translates into an avirulent phenotype in a mouse model for cutaneous leishmaniasis [28]. To determine whether **Δ** *kh1* mutants are compromised in mice, we inoculated the footpads of mice with *Leishmania* promastigotes at stationary phase (5 mice per parasite line), and tracked the infections over a period of 8 weeks. The cohorts of mice inoculated with wild type and **Δ** *kh1*[int*Kh1*] parasites formed 2–3 mm lesions during the 8 week time course (Fig 3A), and the experiment was terminated prior to the generation of open lesions in mice infected with wild type parasites. One mouse out of 5 inoculated with **Δ** *kh1*[int*Kh1*] add-back cells did not cause a detectable lesion, presumably due to a failed injection, and was therefore excluded from this analysis, but the other 4 mice produced lesions of ~2 mm in thickness 8 weeks post infection. In contrast, mice inoculated with **Δ** *kh1* mutant parasites did not generate detectable lesions during this 8-week period. In a separate experiment, we tracked another cohort of 5 mice inoculated with **Δ** *kh1* mutants for 20 weeks and observed a similar failure to elicit lesions (not shown).

Some mutant parasites that do not induce lesions have been observed to persist at the site of infection and thus result in cryptic or subclinical infections [29]. To determine whether **Δ** *kh1* parasites might persist in a cryptic state within mice, we dissected the infected footpads after 8 weeks of infection (Fig 3A) and performed limiting dilution assays to estimate the parasite load in each footpad (Fig 3B). Whereas the estimated parasite burdens caused by wild type and **Δ** *kh1*[int*Kh1*] add-back cells were on average 2.5 x 10^12^ and 1.3 x 10^12^ per lesion, respectively, no parasites could be detected in foot pads from mice inoculated with **Δ** *kh1* mutants (Fig 3B). Together, these results indicate that **Δ** *kh1* mutants, in addition to being unable to cause lesions, are highly compromised and are largely or completely cleared from the tissue of infected mice.

### Δ*kh1* mutants differentiate to axenic amastigotes

**Fig 4:**
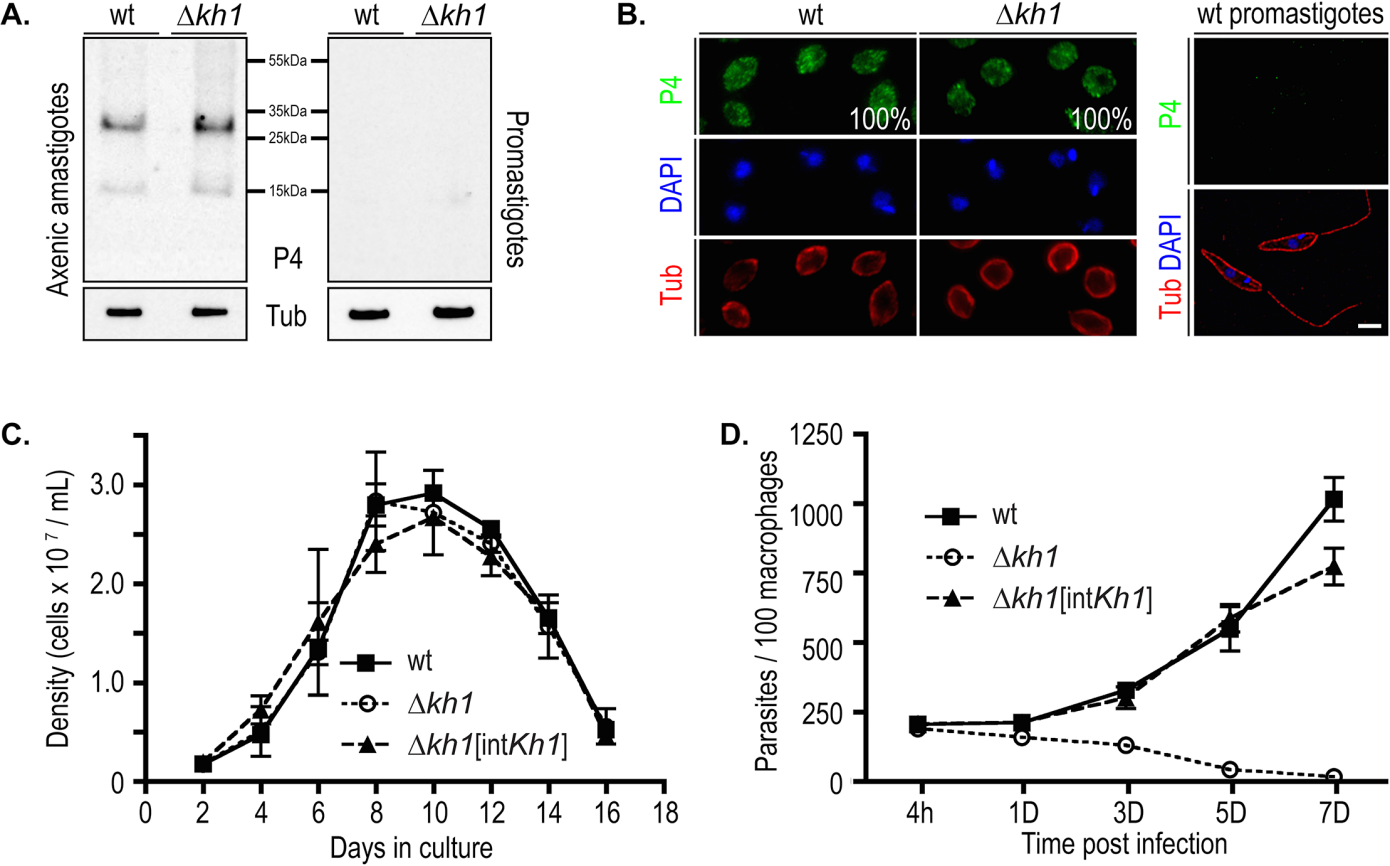
Characterization of Δ*kh1* axenic amastigotes. A. Immunoblot of wild type (wt) and **Δ** *kh1* mutant probed with anti-P4 and anti-**α**-tubulin antibodies as a loading control. The positions of molecular weight markers are indicated. B. Immunofluorescence of the P4 amastigote-specific antigen. Left, axenic amastigotes were stained with anti-P4 antibody (P4, green), DAPI (blue), and anti-**α**-tubulin antibody (Tub, red). The 100% shown in the P4 panel represents the percentage of cells analyzed that are positive for P4 (wt, n = 212; **Δ** *kh1*, n = 240). Right, promastigotes were stained for the same marker as a negative control for the P4 antibody. Scale bar, 3 **μ**m. C. Growth curve of axenic amastigotes comparing growth rate of wild type, **Δ** *kh1*mutants, and **Δ** *kh1*[int*Kh1*] add-back lines over a 16-day time course. Numbers represent the averages and ranges from two independent growth curves (n = 2). Each sample from each growth curve time point was counted in triplicate and the values averaged. D. Quantification of infection of THP-1 macrophages with wild type, **Δ** *kh1*mutants, and **Δ** *kh1*[int*Kh1*] add-back axenic amastigotes. Times post infection: h = hours, D = days. Data represent the averages and standard deviations of triplicate infections (n = 3), each counted in triplicate and averaged. This experiment is similar to that shown in Fig 2E, except that axenic amastigotes, rather than promastigotes, were employed to initiate infection.

Because infection of cultured macrophages [7] and mice (Fig 3) by stationary-phase promastigotes revealed the avirulent nature of **Δ** *kh1* null-mutant parasites, we asked whether this phenotype could be due to **Δ** *kh1* mutants’ inability to differentiate into the infectious mammalian-stage amastigotes. To address this question, we induced the differentiation of promastigotes to axenic amastigotes *in vitro* as previously described [20], and monitored both viability of the axenic amastigotes and the expression of the P4 endonuclease, an amastigote-specific molecular marker [22, 30, 31]. Immunoblot analysis of wild type and **Δ** *kh1* mutants revealed that axenic amastigotes of both lines expressed the same level of the P4 marker (Fig 4A, left panel), but promastigotes did not express this protein (Fig 4A, right panel). Furthermore, all wild type (n = 212) and **Δ** *kh1* mutant (n = 240) parasites examined by immunofluorescence microscopy expressed the P4 marker (Fig 4B). Together, these results indicate that **Δ** *kh1* mutants can successfully differentiate into axenic amastigotes.

Examination over a two-week period revealed no significant growth differences between wild type and **Δ** *kh1* mutant parasites (Fig 4C) in axenic culture, establishing that **Δ** *kh1* mutants are not defective in growth as axenic amastigotes. However, upon infection of cultured THP-1 macrophages with axenic amastigotes, **Δ** *kh1* mutants died over a 7-day time course, whereas wild type and **Δ** *kh1*[int*Kh1*] add-back parasites replicated robustly as intracellular amastigotes (Fig 4D). While these results are similar to those reported previously for infection of macrophages with **Δ** *kh1* promastigotes [7], they make the additional important observation that **Δ** *kh1* mutants that have already differentiated into viable P4-expressing axenic amastigotes, the parasite form used to initiate the macrophage infections in Fig 4D, die when they enter host cells. Hence, lethality of the **Δ** *kh1* null mutant is not the result of failure to differentiate into infectious forms but is rather a phenotype specifically associated with residence within the host macrophage.

### Δ*kh1* mutants show a cytokinesis defect as intracellular amastigotes

To gain a better understanding of how **Δ** *kh1* null mutants are compromised within host cells, we performed a more detailed *in vitro* study by imaging amastigotes inside cultured macrophages during the course of infection. Because **Δ** *kh1* null mutants are rapidly cleared from THP-1 infections, we performed the experiment with a higher multiplicity of infection for each parasite line (10 axenic amastigotes per macrophage) so that there would be enough **Δ** *kh1* parasites to examine while they were being cleared over the infection time course. Consistent with the previous infection using 3 axenic amastigotes per macrophage (Fig 4D), **Δ** *kh1* null mutants were highly compromised and were largely cleared after 7 days (data not shown). Thus, infecting with a higher ratio of parasites to macrophages did not compensate for the avirulent nature of the **Δ** *kh1* null mutants.

**Fig 5:**
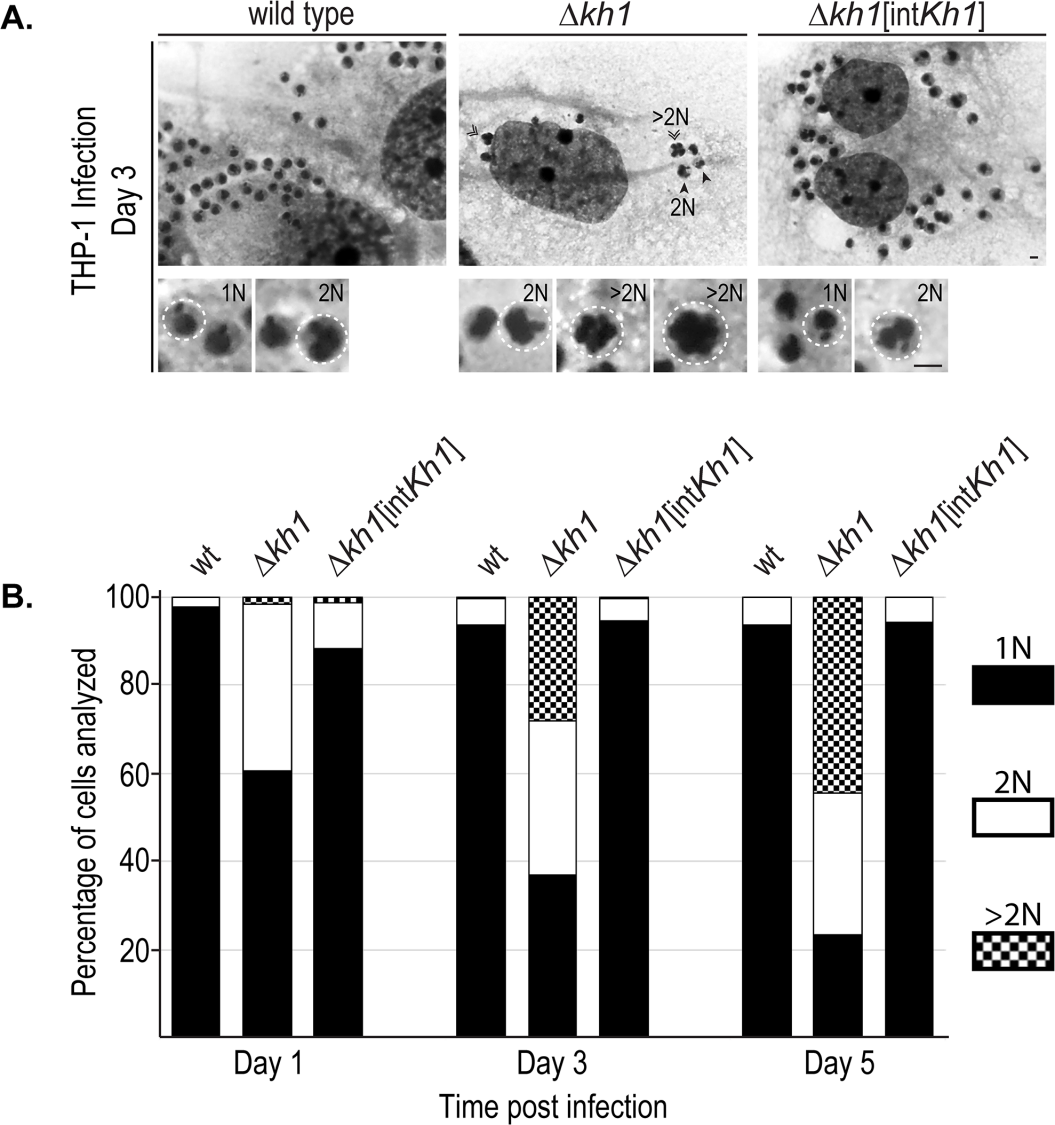
Evidence of multi-nucleated intracellular amastigotes. A. Images of THP-1 cells infected with wild type, **Δ** *kh1*mutants, and **Δ** *kh1*[int*Kh1*] add-back axenic amastigotes at 3 days post infection. For this experiment, a 10:1 ratio of axenic amastigotes to macrophage was used. Multinucleate parasites are indicated by arrowheads (2N) and chevrons (>2N). Bottom, close-up of cells with single or multiple nuclei. N = Nuclei. Scale bars, 3 **μ**m. B. Quantification by microscopy of multi-nucleated intracellular amastigotes. For all time points, the number of intracellular amastigotes examined for each line was >300. Percentages of parasites with 1, 2, and >2 nuclei are indicated as designated by the graph legend at the right. The p-values were calculated for the number of cells with more than two nuclei (>2N) between the different cell lines at Day 5: wt vs. **Δ** *kh1* mutants, p<<0.01; **Δ** *kh1* mutants vs. **Δ** *kh1*[int*Kh1*], p<<0.01; wt vs. **Δ** *kh1*[int*Kh1*], p = 0.8. The p-value calculated for the number of cells with more than two nuclei (>2N) between the different cell lines at Day 5 were as follows: wild type versus **Δ** *kh1* mutants, p<0.01; **Δ** *kh1* mutants versus **Δ** *kh1*[int*Kh1*], p<0.01; wild type versus **Δ** *kh1*[int*Kh1*], p = 0.8.

**Fig 6:**
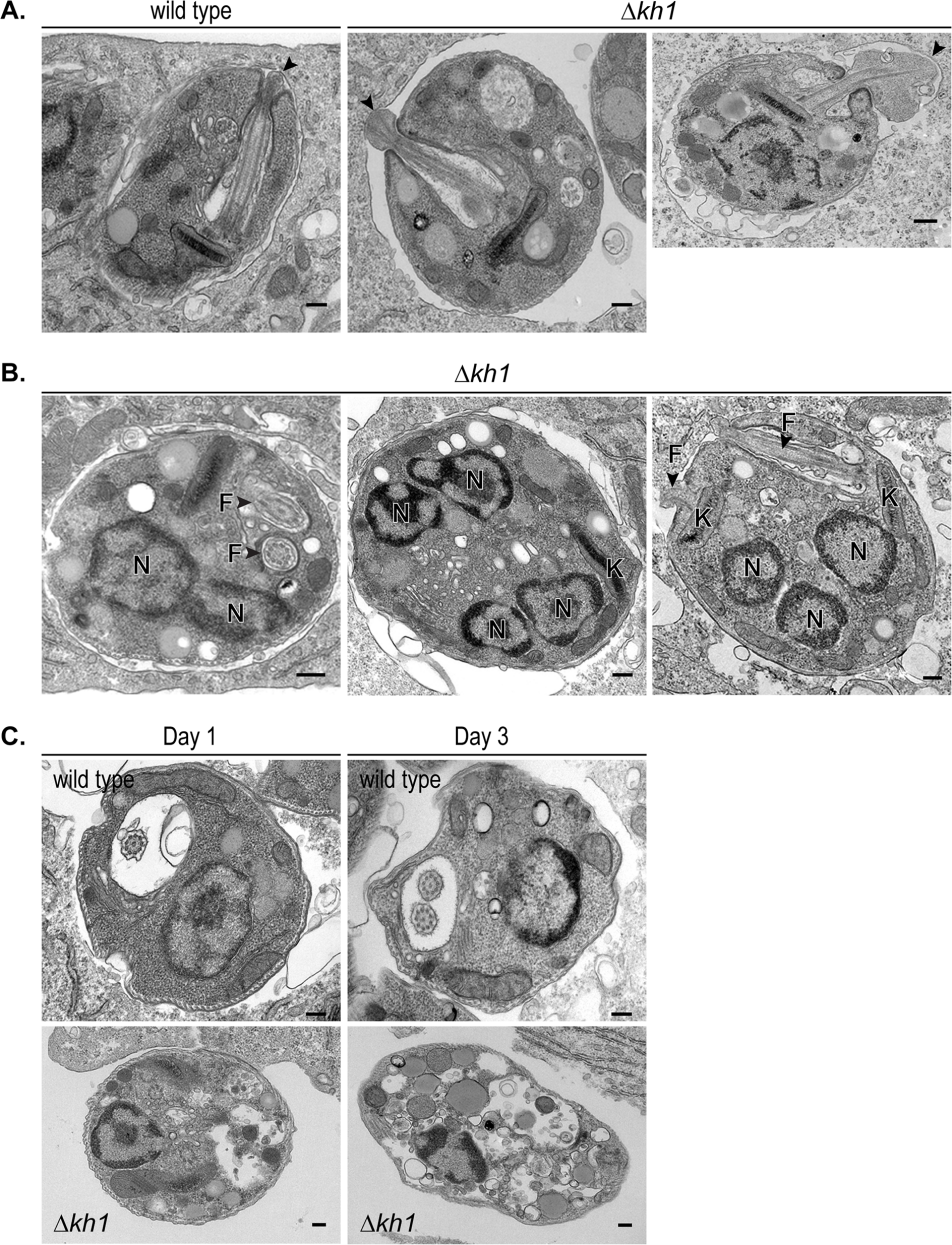
Ultra-structural studies of intracellular amastigotes. **All scale bars represent 200 nm.** A. Morphology of wild type and **Δ** *kh1* amastigotes 1-day post-infection. Arrowheads indicate the tip of the flagellum making contact with the parasitophorous vacuole. B. Multinucleate and multiflagellated **Δ** *kh1* amastigotes inside infected THP-1 macrophages. Nucleus, N; flagellum, F; kinetoplast, K. C. Cellular integrity of wild type and **Δ** *kh1* amastigotes on days 1 and 3 post-infection.

During examination of infected macrophages, we observed a striking phenotype seen only in **Δ** *kh1* mutant parasites. Many of the **Δ** *kh1* mutant amastigotes were multinucleated (Fig 5A) and thus exhibited a larger area of staining than mononucleated amastigotes. Staining and quantification of **Δ** *kh1* amastigotes showed an increased number of cells with multiple nuclei (N) over the infection time course (Fig 5B). At 5 days post-infection, over 70% of **Δ** *kh1* mutant cells examined possessed multiple nuclei (n = 370), with approximately 25% of the population containing 2 nuclei and another 45% containing more than 2 nuclei (>2N). In contrast, we rarely observed >2N cells in the wild type and **Δ** *kh1*[int*Kh1*] populations where >90% of cells examined were 1N (n>400 cells examined per line). Transmission electron micrographs also confirmed the presence of multiple nuclei in **Δ** *kh1* mutant intracellular amastigotes (below, Fig 6B). These results suggest that **Δ** *kh1* mutants are able to replicate DNA and undergo nuclear division, but are unable to execute cytokinesis. Notably, this phenotype is only apparent after **Δ** *kh1* mutants have entered the environment of the host macrophage, and it is not exhibited by **Δ** *kh1* axenic amastigotes.

### Ultrastructural studies reveal morphological differences between wild type and Δ*kh1* intracellular amastigotes and confirm a cytokinesis defect

Previous ultrastructural studies in one of our laboratories (E.G.) on intracellular amastigotes described a putative parasite ‘synapse’ structure between the amastigote flagellum and host parasitophorous vacuole membrane [16]. The authors proposed that this junction might mediate interactions between vacuole membrane ligands and flagellar membrane receptors to promote signal transduction in the parasite and/or serve as a focal point for secretion of parasite proteins into the host cytosol. Should the synapse serve either of these roles, it could be critical for survival of the intracellular amastigotes. Thus, we examined whether the connectivity between the flagellum tip and vacuole membrane is disrupted in **Δ** *kh1* mutants as a possible explanation for the lethal phenotype of this null mutant inside macrophages. The electron micrographs reveal that a ‘synapse’ still forms between the host vacuole membrane and the tip of the flagellum of **Δ** *kh1* parasites (arrowheads in Fig 6A and S1A Fig). However in **Δ** *kh1* parasites, the tip of the flagellum appears enlarged and bulbous compared to the rest of the flagellar axoneme, and in some cases (Fig 6A. **Δ** *kh1*, right most image) the flagellar tip is spectacularly inflated. Similar flagellar morphology is not apparent in wild type or **Δ** *kh1*[int*Kh1*] amastigotes.

In addition to the distorted morphology of the flagellar tip, the shape of the cell body in **Δ** *kh1* amastigotes is abnormal compared to wild type amastigotes. Whereas wild type and **Δ** *kh1*[int*Kh1*] cells normally appear oval-shaped (Fig 6A, left most image and S1 Fig A, right most image), **Δ** *kh1* mutant amastigotes are round (Fig 6A and 6B; S1A Fig). This rounded cell body appears to be specific for the **Δ** *kh1* intracellular amastigotes, as similar ultrastructural analysis of axenic amastigotes revealed no such difference in cell shape between wild type and **Δ** *kh1* mutant parasites (S2 Fig). Furthermore, there were no apparent abnormalities regarding the microtubule structures of the flagellar axoneme and sub-pellicular cytoskeleton in **Δ** *kh1* mutants (S1B and S1C Fig). Cross-sections perpendicular to the flagellar axoneme of **Δ** *kh1* mutant amastigotes appear similar to those of wild type parasites, with the 9V, 9 + 0, and 9 + 2 microtubule arrangements (S1B Fig) observed previously for wild type amastigotes [16]. Similarly, the sub-pellicular microtubule array appears to be unaltered in **Δ** *kh1* null mutant amastigotes (S1C Fig). These results suggest that, other than the overall round cell-shape, there are no observable defects in the gross organization of the flagellar axoneme and sub-pellicular microtubules of **Δ** *kh1* intracellular amastigotes.

Consistent with results reported in Fig 5, many **Δ** *kh1* mutant cells possessed multiple nuclei (>2N) when observed by electron microscopy (Fig 6B). The appearance of separate nuclei suggests that chromosome replication and nuclear separation are unhindered. Furthermore, many **Δ** *kh1* amastigotes possessed two well-separated flagella (Fig 6B). In addition, a large percentage of **Δ** *kh1* amastigotes showed vacuole formation and cellular disintegration (Fig 6C), consistent with the precipitous decline in the number of intracellular amastigotes observed in Fig 2E. Thus, **Δ** *kh1* mutants are able to replicate and separate nuclei and flagella, but they are unable to divide. This defect is specific for the disease-causing intracellular amastigotes and likely accounts for the avirulent nature of **Δ** *kh1* mutant parasites.

## Discussion

This study has focused on the role of the KHARON1 protein in the disease-causing amastigote form of *Leishmania mexicana* parasites. We have shown that KH1 is critical for survival of *L*. *mexicana* intracellular amastigotes and that KH1 is essential for parasite viability within macrophages *in vitro* as well as for virulence in the murine model for cutaneous leishmaniasis. While we do not yet understand the mechanism whereby KH1 supports viability of intracellular amastigotes, several possibilities are worth considering and can guide future studies aimed at elucidating the mechanism of KH1 action.

One set of considerations takes into account important differences between axenic and intracellular amastigotes. The observation that **Δ** *kh1* mutants can differentiate into and survive as extracellular axenic amastigotes that express the amastigote-specific P4 antigen (Fig 4A–4C), but they do not survive as intracellular amastigotes (Fig 4D) indicates that the growth defect and inability to maintain an infection within mammalian hosts exhibited by **Δ** *kh1* mutants is specific to intracellular amastigotes once they take up residence in host cells. A potential explanation for the selective requirement for KH1 in intracellular amastigotes is that KH1 may mediate an essential interaction between the intracellular parasite and its host cell. Thus, a plausible hypothesis is that proper formation of the flagellar membrane of amastigotes, e.g., targeting of proteins required for proper sensory or secretory function, is essential for these parasites to survive in the hostile environment of the macrophage phagolysosome. Alternatively, the critical requirement for KH1 may only emerge once amastigotes have become fully differentiated inside the macrophage and not be apparent in axenic amastigotes, which differ in gene expression from intracellular amastigotes and may represent an intermediate stage between promastigotes and true amastigotes [32].

Next, we consider the speculated role of KHARON1 in targeting proteins that may be involved in ‘synapse’ formation between the tip of the amastigote flagellum and the macrophage parasitophorous vacuole. KH1 is important for trafficking of the *L*. *mexicana* Glucose Transporter 1 (LmxGT1) to the flagellar membrane of promastigotes. However, LmxGT1 expression cannot be detected during the amastigote stage of the parasite life cycle [13], and thus the lethal phenotype of **Δ** *kh1* mutants inside macrophages is unlikely to be due to failure to traffic LmxGT1 to the amastigote flagellum. We have shown in this study that KH1 is indeed expressed in amastigotes and is localized to the internal flagellum and pellicular cytoskeleton (Fig 1). Additional ultrastructural studies (Fig 6A) indicated that the connectivity between the flagellum tip and vacuole membrane is not obviously disrupted in **Δ** *kh1* amastigotes. However, the morphology of the flagellar tip is clearly distorted in **Δ** *kh1* compared to wild type amastigotes, thus raising the possibility that the putative synapse could experience functional aberrations in **Δ** *kh1* null mutants, even though the structure is not massively disrupted. It has been speculated that the synapse could be involved in either sensing of signals from the macrophage or delivery of possible effector proteins to the host cell [16], and disruption of these functions could result in parasite death following entry into the host cell.

Another issue to consider is that KH1 resides in at least two locations within amastigotes, the flagellum and the subpellicular cytoskeleton. The critical role of KH1 for amastigote viability could be executed at either one or both of these subcellular locations. Flagella have been identified as essential mediators of cytokinesis in infectious bloodstream form African trypanosomes, kinetoplastid parasites related to *Leishmania* species. In trypanosomes proper flagellar function, including attachment of the nascent flagellum to the parental flagellum during flagellar extension, appears to be required for positioning the cleavage furrow that initiates cytokinesis. Hence, RNAi induced disruption of the expression of many flagellar proteins in *Trypanosoma brucei* resulted in failed cytokinesis and ‘monster cells’ exhibiting multiple nuclei and flagella [8]. However, it is difficult to imagine how the short, non-motile amastigote flagellum could be similarly required for cytokinesis of *Leishmania* amastigotes. An alternative explanation for the lethal phenotype of **Δ** *kh1* null mutants in intracellular amastigotes is that the presence of KH1 in the subpellicular microtubules (Fig 1C and 1E) is essential for morphogenetic changes associated with initiation of cytokinesis in these intracellular amastigotes. During division, *Leishmania* promastigotes adopt a rounded cell-shape after DNA segregation but prior to cytokinesis [33, 34], and remodeling of the subpellicular cytoskeleton might also be required for cell division of amastigotes. The experimental observation of multinucleated, spherical-shaped **Δ** *kh1* mutant intracellular amastigotes that are unable to divide suggests that deletion of the *Kh1* genes might induce cytoskeletal defects that contribute to failure of cytokinesis.

In summary, the lethal effect of deleting the *Kh1* genes on intracellular amastigotes could be due to i) loss of KH1 function at the base of the flagellum, ii) elimination of KH1 activity at the subpellicular microtubules, or iii) depletion of KH1 at both sites. In the future, distinguishing between these possibilities may be aided by preliminary observations suggesting that KH1 interacts with other partner proteins and that a subset of the interacting partners are specific for either the subpellicular cytoskeleton or the base of the flagellar axoneme (Khoa Tran, unpublished results). These observations suggest that there could be at least two separate KHARON Complexes, one complex functioning at the subpellicular cytoskeleton and the other at the base of the flagellum. Targeted gene replacement of specific genes encoding selectively localized partner proteins may allow the selective destruction of each of the two hypothesized KHARON Complexes, thus elucidating the function of KH1 at each subcellular location.

This study emphasizes the importance of KH1 in the cell biology and virulence of *Leishmania* parasites. Ongoing studies on the *T*. *brucei* ortholog of KH1 also indicate that this protein is important for the flagellar targeting of a putative Ca^2+^ channel, and is also essential for viability of both bloodstream and procyclic forms of that parasite (Marco Sanchez, unpublished results). Hence, KH1 plays an essential role in both intracellular *Leishmania* amastigotes and extracellular African trypanosomes, underscoring its vital function in the biology of several kinetoplastid protozoa with distinct modes of parasitism.

## Supporting Information

S1 Fig Flagellar and microtubule ultra-structures of intracellular Δ*kh1* amastigotes. A. Examples of the flagellar tip (indicated by arrowheads) from **Δ** *kh1* and **Δ** *kh1*[int*Kh1*] amastigotes. Nucleus, N; flagellum, F; kinetoplast, K. Scale bars represent 200 nm. B. Cross-sections of the flagellum in wild type and **Δ** *kh1* amastigotes showing 9+2, 9+0, and 9V (V = variable) axoneme structure. Scale bars represent 50 nm. C. Sub-pellicular microtubule network in **Δ** *kh1* and **Δ** *kh1*[int*Kh1*] amastigotes. Arrowheads in the expanded images at the bottom indicate sub-pellicular microtubules. Scale bars represent 200 nm.
(TIFF)

S2 Fig Ultra-structure of wild type and Δ*kh1* mutant axenic amastigotes. Arrows indicate flagellar tip. Scale bar represents 1**μ**m.
(TIFF)
