# Supplementary figures and images for: Kharon1 Null Mutants of Leishmania mexicana Are Avirulent in Mice and Exhibit a Cytokinesis Defect within Macrophages
Source: PLoS One. 2015 Aug 12;10(8):e0134432. doi: 10.1371/journal.pone.0134432 (PMC4534133; doi:10.1371/journal.pone.0134432)

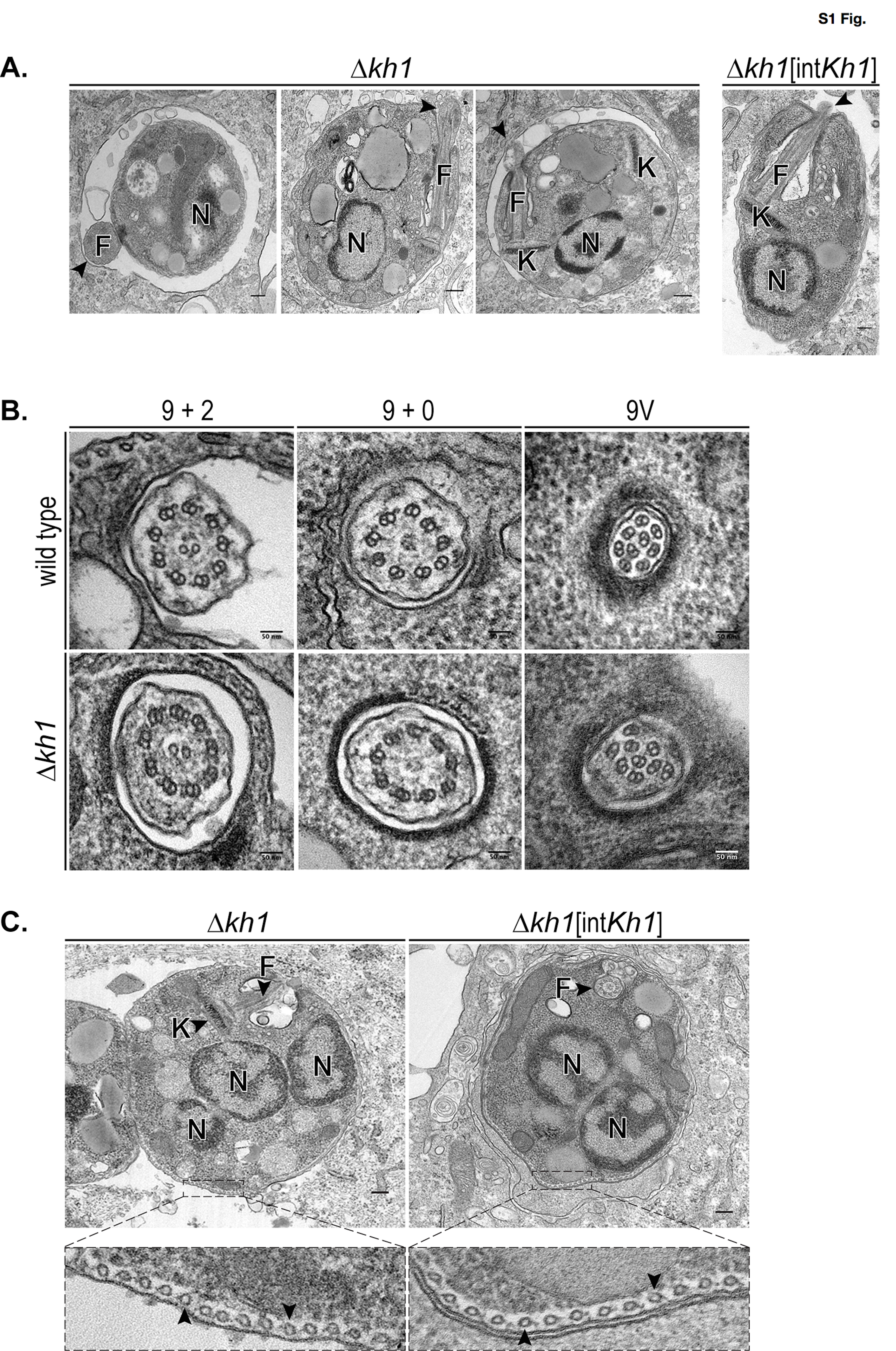

Supplement: S1 Fig — A. Examples of the flagellar tip (indicated by arrowheads) from Δ kh1 and Δ kh1[intKh1] amastigotes. Nucleus, N; flagellum, F; kinetoplast, K. Scale bars represent 200 nm. B. Cross-sections of the flagellum in wild type and Δ kh1 amastigotes showing 9+2, 9+0, and 9V (V = variable) axoneme structure. Scale bars represent 50 nm. C. Sub-pellicular microtubule network in Δ kh1 and Δ kh1[intKh1] amastigotes. Arrowheads in the expanded images at the bottom indicate sub-pellicular microtubules. Scale bars represent 200 nm. (TIFF) [file pone.0134432.s001.tiff]

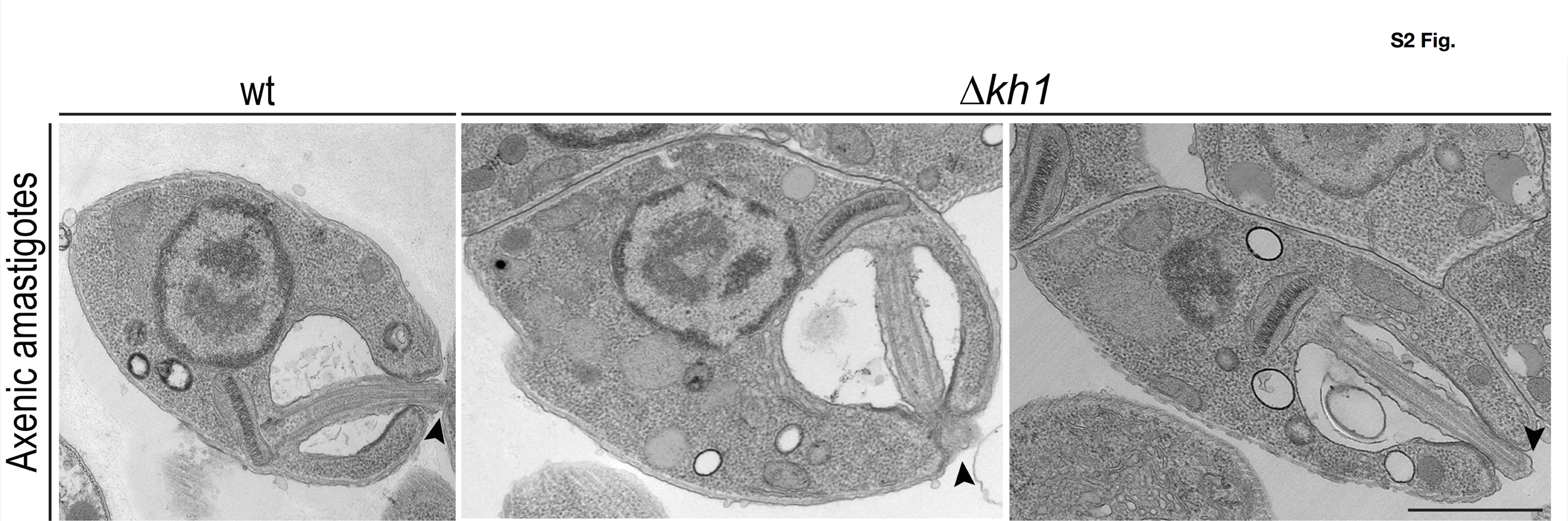

Supplement: S2 Fig — Arrows indicate flagellar tip. Scale bar represents 1μm. (TIFF) [file pone.0134432.s002.tiff]
